# Nut consumption in a representative survey of Australians: a secondary analysis of the 2011–2012 National Nutrition and Physical Activity Survey

**DOI:** 10.1017/S1368980019004117

**Published:** 2020-12

**Authors:** Cassandra J Nikodijevic, Yasmine C Probst, Marijka J Batterham, Linda C Tapsell, Elizabeth P Neale

**Affiliations:** 1 Faculty of Science, Medicine and Health, School of Medicine, University of Wollongong, Wollongong, NSW 2522, Australia; 2 Illawarra Health and Medical Research Institute, University of Wollongong, Wollongong, NSW 2522, Australia

**Keywords:** Nut consumption, National Nutrition and Physical Activity Survey, Obesity, Australia

## Abstract

**Objective:**

Nut consumption is associated with a range of health benefits. The current study aimed to examine nut consumption in the 2011–2012 National Nutrition and Physical Activity Survey (NNPAS) and to investigate associations between nut intake, nutrient intake and anthropometric and blood pressure measurements.

**Design:**

Secondary analysis of the 2011–2012 NNPAS. Usual consumption of nuts in the 2011–2012 NNPAS was determined, and nut consumption was compared with population recommendations of 30 g nuts per day. The relationship between nut consumption and intakes of key nutrients, anthropometric outcomes (weight, BMI and waist circumference) and blood pressure was examined using linear regression for participants aged over 18 years.

**Setting:**

Australia.

**Participants:**

Australians (2 years and older, *n* 12 153) participating in the representative 2011–2012 NNPAS.

**Results:**

Mean nut intake was 4·61 (95 % CI: 4·36, 4·86) g/d, with only 5·6 % of nut consumers consuming 30 g of nuts per day. Nut consumption was associated with significantly greater intakes of fibre, vitamin E, Fe, Mg and P. There was no association between nut consumption and body weight, BMI, waist circumference, or blood pressure.

**Conclusions:**

Exploration of nut consumption in a representative sample of Australians identified that nut intake does not meet recommendations. Higher nut consumption was not adversely associated with higher body weight, aligning with the current evidence base. Given the current levels of nut consumption in Australia, strategies to increase nut intake to recommended levels are required.

Habitual consumption of nuts has been associated with reduced risk of a range of chronic diseases such as CVD^(1)^, type 2 diabetes mellitus^(2)^ and metabolic syndrome^(3)^. Research also suggests that individuals who consume nuts tend to gain less weight over time^(4)^ and may have healthier diets overall^(5–8)^. As a result, regular nut consumption (approximately 30 g/d) is recommended by numerous dietary guidelines globally^(9)^. Estimations of population nut consumption are required to assess compliance with guidelines and guide strategies to promote nut intake.

Results from the National Nutrition and Physical Activity Survey (NNPAS) component of the 2011–2013 Australian Health Survey (AHS) suggest that on the day of the survey, Australians consumed a mean of 5·2 g of nuts, considerably below the recommended 30 g^(10)^. However, this information did not include nut consumption from mixed dishes such as breakfast cereals, baked goods or muesli bars^(10)^, which may underestimate total nut consumption. A national survey conducted in New Zealand^(11)^ considered whole nuts, nut butters and nuts contained in mixed dishes, such as breakfast cereals and muesli bars. Approximately, 19 % of the mean nut intake of this population was derived from nuts present in mixed dishes^(11)^, suggesting these products are substantial contributors to nut intake. To accurately estimate nut consumption, a systematic method of quantifying the amount of nuts in mixed dishes is required. We have recently developed a nut-specific database for application to Australian dietary data^(12)^. In addition, currently available data on nut consumption from the 2011–2012 NNPAS are based on a single day’s dietary recall. As nuts are likely to be an episodically consumed food, relying on an individual day of dietary data to quantify intake may result in inaccurate estimations of intake^(13)^.

Although nut consumption is associated with a range of health benefits, the limited population intake data currently available for Australia reduce our capacity to confirm favourable health outcomes in the Australian context. There is a need to explore the relationship between nut consumption and health outcomes in a representative Australian sample. The aim of the current study was to perform a secondary analysis of the 2011–2012 NNPAS, within the 2011–2013 AHS, to quantify nut consumption in Australia and identify associations between nut intake and anthropometric and blood pressure measurements. A secondary aim was to identify the contribution of nuts to intakes of key nutrients.

## Methods

The 2011–2012 NNPAS was a national nutrition survey conducted with 12 153 Australians aged 2 years and older^(14)^. The details of the 2011–2012 NNPAS have been published previously^(14)^; however, briefly, the 2011–2012 NNPAS was a component of the 2011–2013 AHS. The 2011–2013 AHS sampled individuals from private dwellings in Australia. Individuals residing in non-private dwellings such as boarding schools, prisons, hospitals and nursing homes were excluded from the survey^(14)^. Private dwellings were selected for inclusion in the survey based on a stratified multistage area sample. Within the dwellings, one adult and one child (where appropriate) were selected for inclusion in the survey. Household and initial person weights were calculated to ensure that results were representative of the total population. These weights were then benchmarked to the estimated population residing in private dwellings^(14)^.

A total of 12 336 households were approached for inclusion in the survey, with 9519 households included in the survey (77 % response rate). This resulted in the total of 12 153 participants included in the survey^(14)^.

The 2011–2012 NNPAS consisted of two 24-h recalls conducted on two separate days – the first 24-h recall was completed in person by all participants, with a second 24-h recall completed via telephone at least 8 d after the first interview. Follow-up contact phone details to complete the second 24-h recall were requested for all participants, which was conducted in approximately 63 % of participants^(14)^. The 24-h recalls collected data about all foods and beverages consumed the day prior to the survey. The 24-h recall used the Automated Multiple-Pass Method^(15)^ for data collection, which was adapted to align with the Australian food supply. The Automated Multiple-Pass Method used involved five phases, the details of which are outlined elsewhere^(14)^.

The current analysis utilised the Confidentialised Unit Record Files (CURF) from the 2011–2012 NNPAS. The authors registered with the Australian Bureau of Statistics (ABS) to gain permission to access the 2011–2012 NNPAS data for research purposes. The Census and Statistics Act 1905 allows access to the 2011–2012 NNPAS.

In the current study, data on intake of foods and corresponding nutrient intakes from both 24-h recalls were used. Dietary data were analysed using the AUSNUT 2011–2013 food composition database, which uses a nested hierarchical structure of major, sub-major and minor food groups^(16)^. Foods were categorised as discretionary or non-discretionary using the AHS Discretionary Food List^(17)^.

In addition to dietary data, measured body weight, BMI, waist circumference and blood pressure were also used. In the 2011–2012 NNPAS, body weight was recorded in kilograms to one decimal place, using a digital scale^(14)^. Height was measured using a stadiometer, in centimeters to one decimal place. BMI was calculated as weight (kg) divided by height (m) squared. Waist circumference was taken at the top of the belly button and was recorded in centimeters to one decimal place. In the case of waist circumference and height, a second measurement was taken on a random 10 % sample. In the case that there was more than 1 cm variation, a third measurement was taken^(14)^. Systolic and diastolic blood pressure measurements were taken on participants over 5 years of age using an automated blood pressure monitor. Two measurements were taken on each participant. In the case that there was more than a 10 mmHg difference between the measurements, a third measurement was taken. In addition, demographic characteristics including age, sex, education level and physical activity level were also used. In the 2011–2012 NNPAS, the highest level of non-school educational attainment was self-reported by participants aged 15 years and over^(14)^. Physical activity information was collected for participants aged 18 years and over by asking for self-reported time spent and frequency of walking for transport or for fitness, moderate physical activity and vigorous physical activity. This information was then categorised into levels of physical activity, which are outlined in further detail elsewhere^(14)^.

To determine nut consumption during the 2011–2012 NNPAS, the nut-specific database developed by our team^(12)^ was applied to 24-h recall data. For the purpose of this research, the term ‘nuts’ was used to include tree nuts, peanuts, mixed nuts, tree nut products and peanut products and excluded coconut, coconut products, peanut oil, tree nut oils and nut milks. Nuts and nut-containing products were identified, as well as the percentage and type of nuts in each product. The percentage of a product containing nuts (nut content) was multiplied by the total weight of the product to determine the amount of nuts (in grams) consumed by each participant for each product. Nut consumption reported in the first and second 24-h recall was then determined.

The multiple source method (MSM) was used to calculate usual intake of nuts. Details of the MSM model have been published previously^(18)^; briefly, the MSM model uses logistic regression models to determine the individual probability of consumption of a food or nutrient and the estimation of the consumption amount. These models were then applied to estimate usual consumption.

### Statistical analysis

Analysis of usual nut consumption was conducted using Stata/IC Version 15 (version 15, StataCorp, 2017). Following the instructions of the ABS, the sampling weights and sampling design of the 2011–2013 AHS were accounted for during the data analysis by applying replicate weights^(14)^. Population weights were applied to ensure data were representative of the larger Australian population. Commands were performed in Stata using the complex design model^(19)^ to account for sampling design to calculate means (reported as mean and 95 % CI), median (25th and 75th percentiles) and proportions (reported as a percentage) of interest, as outlined below. Mean and 95 % CI and median and 25th and 75th percentiles for nut consumption (as g/d) were determined for all participants. The level of nut consumption was also presented across quartiles of intake, which were also calculated using the complex design model. Participants were categorised into subsamples as either ‘nut consumers’ or ‘non-consumers’ of nuts, and the proportion of ‘nut consumers’ and ‘non-consumers’ was determined. Nut intake was examined according to the proportion of individuals consuming 5-g increments for both all participants and for the subsample of ‘nut consumers’. The proportion of Australians and ‘nut consumers’ meeting the recommended 30 g of nuts per day was calculated, as well as the proportion consuming over 42·5 g of nuts per day and 60 g of nuts per day, to align with other recommendations identified in previous research^(20,21)^. To examine nut intake in different population groups, the mean nut intakes in both Australians and ‘nut consumers’ (as g/d), as well as nut consumption by age, sex and education categories, were then examined. Nut consumption was compared across age and sex categories and education categories using linear regression with pairwise comparisons of marginal linear predictions with Bonferroni adjustment within each variable (using the number of comparisons as the denominator).

The relationship between nut intake and consumption of key nutrients was explored via survey-adjusted linear regression. Linearity, homoscedasticity and normality of residuals were checked for all variables for all regression analyses conducted. Nut intake was explored as g/d. The key nutrients examined were dietary fibre, vitamin E, Ca, Fe, Mg and P. The selection of nutrients was based on those nutrients found to be higher in ‘nut consumers’ in a representative sample in New Zealand^(8)^. To facilitate the current analysis, usual intake of key nutrients and dietary energy was calculated using MSM. Covariates included in the analysis were age, sex, usual energy intake (kJ/d), physical activity (ranging from ‘sedentary’ to ‘high’) and education level. As education level is defined as level of non-school education, data on participants who were currently attending school (i.e. children aged less than 18 years) were excluded from all regression analyses. Participants with missing data for any of the variables (e.g. missing data for physical activity) were also excluded from the subsequent regression analyses.

Linear regression was performed to explore the association between nut consumption and anthropometric measures (weight, BMI, waist circumference) and systolic and diastolic blood pressure. As with the nutrient analysis, nut consumption was presented as grams of nut intake, and covariates used were age, sex, usual energy intake (kJ/d), physical activity (ranging from ‘sedentary’ to ‘high’) and education level. In addition, for blood pressure analyses, usual intakes of Na and K were included in the analysis, due to the association of these nutrients with blood pressure^(22,23)^. BMI was also included as a covariate in the blood pressure analyses, to account for variations in blood pressure due to body mass. To explore causality in these models, directed acyclic graphs were developed using DAGitty^(24)^.

### Exploration of food group sources of nuts

The proportion of nuts (presented as a percentage) contributed by AUSNUT 2011–2013 major and sub-major food groups was determined. To provide an overview of contribution to food group and nutrient intakes, the current analysis was restricted to data from Day 1 of the 2011–2012 NNPAS only, as outlined previously in analyses examining other dietary components in the 2011–2012 NNPAS^(25)^. Furthermore, the analysis was restricted to ‘nut consumers’ only to avoid skewing the results.

The proportion of nuts (presented as a percentage) contributed by AUSNUT 2011–2013 major and sub-major food groups was then determined. In addition, the amount of nuts consumed as discretionary and non-discretionary products was first quantified and then calculated as a proportion of total nut intake. The types of nuts consumed were also calculated as a proportion of total nut intake. This was based on categorisation of nut type in the nut-specific database.

## Results

### Proportions of ‘nut consumers’ (Day 1 and Day 2 data) and mean nut intake

From the 2011–2012 NNPAS results, 39·2 % of Australians were found to be ‘nut consumers’ and 60·8 % were ‘non-consumers’. Among Australians, the mean nut intake was 4·61 (95 % CI: 4·36, 4·86) g/d. Median nut consumption among Australians was 0·00 (25th and 75th percentiles: 0·00–6·27) g/d.

When examined for all Australians (including those who did not consume nuts), the proportion of Australians who were consuming the recommendation of 30 g or more per day was 2·2 %, leaving 97·8 % of the population not meeting the recommendation. Table 1 shows the proportions of all Australians consuming various amounts of nuts per day. When the analysis was limited to ‘nut consumers’, the mean nut intake was 11·75 (95 % CI: 11·30, 12·20) g/d, with a median intake of 8·85 (25th and 75th percentiles: 4·78–14·94) g/d. The proportion of ‘nut consumers’ meeting the recommendation of 30 g or more per day was 5·6 %; therefore, 94·4 % of ‘nut consumers’ were having less than 30 g of nuts per day. Amongst those who met the 30 g/d recommendation, considerably smaller proportions met the higher consumption cut-offs of 42·5 and 60 g/d (Table 1).

**Table 1:** Percentage of Australians and ‘nut consumers’ reporting different levels of nut intake, 2011–2012 National Nutrition and Physical Activity Survey (NNPAS)*

Amount of nuts consumed per day (g)	Australians		Nut consumers	
	Percentage	95 % CI	Percentage	95 % CI
0	60·78	59·46, 62.10	–	–
<5	10·42	9·61, 11·23	26·57	24·54, 28·60
5 to <10	12·38	11·60, 13·16	31·56	29·82, 33·31
10 to <15	6·64	5·94, 7·35	16·94	15·40, 18·49
15 to <20	3·09	2·66, 3·52	7·88	6·79, 8·97
20 to <25	3·18	2·77, 3·58	8·10	7·07, 9·12
25 to <30	1·32	1·03, 1·61	3·36	2·65, 4·07
30 to <42·5	1·48	1·09, 1·86	3·76	2·79, 4·73
42·5 to <60	0·45	0·30, 0·60	1·14	0·76, 1·52
60 or more	0·27	0·16, 0·38	0·69	0·42, 0·96

* Australians: population size: 21 526 456, number of observations: 12 153; nut consumers: population size: 8 441 642, number of observations: 4765.

Nut consumption was also examined according to age and sex groups. Nut consumption amongst males and females by age category (for both all Australians and ‘nut consumers’ only) is shown in Table 2. For Australian males, adults aged 18–64 years reported the highest nut consumption (mean: 5·42 g (95 % CI: 4·94, 5·89)), which was significantly higher than intakes in male children (*P* < 0·001, *P* < 0·001, *P* = 0·001 for adults *v*. children aged 2–8 years, 9–13 years and 14–17 years, respectively, Table 2). There were no significant differences between older male adults and older male children (aged 14–17 years) and older male adults and males aged 18–64 years. Similar patterns of intake were found for Australian females, with the highest nut intakes in adults aged 18–64 years (mean: 5·19 g (95 % CI: 4·79, 5·60)). When analyses were limited to ‘nut consumers’, nut intakes were also highest amongst adults aged 18–64 years (males: mean 13·40 g (95 % CI 12·60, 14·19), females: mean 12·31 g (95 % CI 11·62, 13·00)). For male ‘nut consumers’, those aged 18–64 years consumed significantly higher amounts of nuts than children (*P* < 0·001, *P* = 0·001, *P* = 0·002 for adults *v.* children aged 2–8 years, 9–13 years and 14–17 years, respectively, Table 2), with no significant differences between males aged 18–64 years and older males or older males and children aged 9 years and older. Results were similar for female ‘nut consumers’, although female ‘nut consumers’ aged 18–64 years were found to consume significantly higher amounts of nuts than all other age groups, including older adults (*P* < 0·001, *P* < 0·001, *P* < 0·001, *P* = 0·004 for adults *v*. children aged 2–8 years, 9–13 years and 14–17 years, and older adults, respectively, Table 2). Table 3 shows the amount of nut consumption according to education levels, respectively. Among all Australians, individuals with higher education levels had the highest consumption of nuts (mean: 6·75 g (95 %CI 5·67, 7·82) for individuals with the highest level of education, in comparison with mean: 4·12 g (95 % CI 3·75, 4·50) for individuals with no non-school qualification). When examined via formal statistical tests, it was found that the top two levels of education had significantly higher nut intake than the lower two levels (*P* < 0·001 for no non-school qualification *v*. bachelor degree/diploma, and *v*. postgraduate degree/graduate diploma, *P* < 0·001, *P* = 0·003 for certificate *v.* bachelor degree/diploma, and postgraduate degree/graduate diploma, respectively, Table 3). When the current analysis was limited to ‘nut consumers’, however, intakes were similar between education levels (Table 3). Patterns of nut intake according to quartiles of nut consumption are shown in Table 4.

**Table 2:** Mean (se) and median (25th and 75th percentiles) nut consumption by sex and age groups, for all Australians, and ‘nut consumers’ only, 2011–2012 National Nutrition and Physical Activity Survey (NNPAS)*

Sex and age group	All Australians						Nut consumers					
	Population size	Number of observations	Mean	(95 % CI) nut intake (g)	Median	(25th and 75th percentiles) nut intake (g)	Population size	Number of observations	Mean	(95 % CI) nut intake (g)	Median	(25th and 75th percentiles) nut intake (g)
Males												
Children (2–8 years)	1 002 059	625	2·51	1·95, 3·07^a^	0	0–3·56	352 381	223	7·13	5·92, 8·34^a^	5·60	3·15–8·94
Children (9–13 years)	770 918	392	2·79	1·95, 3·63^a^	0	0–2·72	244 494	119	8·80	6·77, 10·83^a,b^	6·32	3·30–10·51
Children (14–17 years)	528 535	356	3·05	2·11, 3·98^a,b^	0	0–3·94	172 324	123	9·35	7·58, 11·11^a,b^	6·40	3·95–13·75
Adults (18–64 years)	7 031 494	3419	5·42	4·94, 5·89^c^	0	0–7·49	2 842 309	1352	13·40	12·60, 14·19^c^	9·63	5·42–17·86
Older adults (65 years and over)	1 374 767	910	4·62	3·77, 5·47^b,c^	0	0–5·88	508 022	351	12·50	10·86, 14·14^b,c^	9·18	4·67–18·28
Females												
Children (2–8 years)	931 442	628	1·61	1·22, 2·01^a^	0	0–1·78	254 363	167	5·91	4·97, 6·85^a^	4·82	2·68–7·50
Children (9–13 years)	755 089	395	2·36	1·67, 3·05^a,b^	0	0–2·69	229 460	129	7·77	6·03, 9·50^a,b^	5·11	3·00–9·17
Children (14–17 years)	496 206	322	3·29	2·48, 4·10^b,c^	0	0–5·59	191 970	121	8·51	7·35, 9·66^b^	8·29	4·85–10·08
Adults (18–64 years)	7 090 526	3913	5·19	4·79, 5·60^d^	0	0–7·89	2 991 184	1682	12·31	11·62, 13·00^c^	9·47	5·42–15·71
Older adults (65 years and over)	1 545 421	1193	4·35	3·78, 4·93^c,d^	0	0–6·38	655 134	498	10·26	9·48, 11·05^b^	7·46	4·51–14·14

^a,b,c,d^Indicate significant differences (*P* < 0·05) between groups after Bonferroni adjustment.

* Australians: population size: 21 526 456, number of observations: 12 153; nut consumers: population size: 8 441 642, number of observations: 4765.

**Table 3:** Mean (se) and median (25th and 75th percentiles) nut consumption by education level, for all Australians, and ‘nut consumers’ only, 2011–2012 National Nutrition and Physical Activity Survey (NNPAS)*

Education level	All Australians						Nut consumers					
	Population size	Numbers of observations	Mean	(95 % CI) nut intake (g)	Median	(25th and 75th percentiles) nut intake (g)	Population size	Numbers of observations	Mean	(95 % CI) nut intake (g)	Median	(25th and 75th percentiles) nut intake (g)
Postgraduate Degree, Graduate Diploma/Graduate Certificate	1 307 196	770	6·75	5·67, 7·82^a^	3·53	0–10·41	749 325	436	11·77	10·20, 13·34^a^	9·00	4·76–14·58
Bachelor Degree, Advanced Diploma/Diploma	4 687 485	2604	6·32	5·87, 6·78^a^	0	0–9·68	2 310 840	1267	12·83	12·13, 13·53^a^	9·74	5·66–17·22
Certificate (Certificate III/IV, Certificate I/II, Certificate not further defined)	4 241 392	2263	4·72	4·19, 5·24^b^	0	0–6·09	1 533 592	845	13·05	11·95, 14·14^a^	9·51	5·17–17·85
No non-school qualification	7 339 153	4190	4·12	3·75, 4·50^b^	0	0–5·39	2 555 554	1465	11·84	10·95, 12·74^a^	8·95	4·90–14·72

^a,b^Indicate significant differences (*P* < 0·05) between groups after Bonferroni adjustment.

* Australians: population size: 17 575 226, number of observations: 9827; nut consumers: population size: 7 149 311, number of observations: 4013.

**Table 4:** Nut consumption by quartiles, 2011–2013 National Nutrition and Physical Activity Survey (NNPAS) (population size: 21 526 456, number of observations: 12 153)

	Non-consumers	Nut consumers			
		Quartile 1	Quartile 2	Quartile 3	Quartile 4
Minimum–maximum nut intakes (g)	0	>0–4·78	4·79–8·85	8·86–14·94	≥14·95
Percentage of individuals	60·8	9·8	9·8	9·8	9·8
Nut intake (g)					
Mean	0	3·03	6·59	11·30	26·07
95 % CI		2·96, 3·10	6·50, 6·68	11·14, 11·46	25·27, 26·88
Median	0	3·07	6·45	10·91	22·33
25th and 75th percentiles	0·0	2·16–3·99	5·65–7·48	9·68–12·79	19·05–29·12

### Association between nut intake and nutrient intakes (Day 1 and Day 2 data)

The relationship between nut intake and consumption of key nutrients was explored in participants aged 18 years and older. The proposed causal model underlying the current analysis is shown in online supplementary material (Supplemental Fig. 1). After adjusting for covariates, greater consumption of nuts was associated with significantly higher consumption of fibre (g) (coefficient per gram of nuts: 0·09, 95 % CI: 0·07, 0·11, *P* < 0·001), vitamin E (mg) (coefficient: 0·07, 95 % CI: 0·06, 0·08, *P* < 0·001), Fe (mg) (coefficient: 0·01, 95 % CI: 0·01, 0·02, *P* = 0·001), Mg (mg) (coefficient: 1·83, 95 % CI: 1·57, 2·08, *P* < 0·001) and P (mg) (coefficient: 0·71, 95 % CI: 0·06, 1·35, *P* = 0·032) (see online supplementary material, Supplemental Tables 2–6). Non-significant associations were found between nut consumption and Ca (mg) (see online supplementary material, Supplemental Table 7).

### Associations between nut consumption and health measures (Day 1 and Day 2 data)

The relationship between nut intake and health measures was explored in participants aged 18 years and older. The proposed causal model underlying the current analysis is shown in online supplementary material (Supplemental Figs. 8 and 9). When adjusted for covariates, consumption of nuts was not significantly associated with increased body weight (kg) (coefficient per gram of nuts: −0·01, 95 % CI: −0·06, 0·05, *P* = 0·823), BMI (kg/m^2^) (coefficient: −0·01, 95 % CI: −0·02, 0·01, *P* = 0·390) or waist circumference (cm) (coefficient: −0·03, 95 % CI: −0·07, 0·01, *P* = 0·181) (see online supplementary material, Supplemental Tables 10–12). Consumption of nuts was not significantly associated with systolic and diastolic blood pressure (mmHg), when adjusted for covariates (see online supplementary material, Supplemental Tables 13 and 14).

### Food group sources of nut intake (Day 1 data only)

The AUSNUT 2011–2013 major food groups that contributed towards the nut intake of ‘nut consumers’ included ‘Seed and nut products and dishes’ (76·08 %), ‘Confectionery and cereal/nut/fruit/seed bars’ (7·33 %), ‘Cereals and cereal products’ (5·21 %) and ‘Cereal-based products and dishes’ (3·26 %) (Table 5).

**Table 5:** Contribution of major and sub-major food groups towards the mean nut intake of ‘nut consumers’, 2011–2012 National Nutrition and Physical Activity Survey (NNPAS) (number of observations: 3761)*

Major food group	Percentage of nut intake of ‘nut consumers’
Seed and nut products and dishes	**76·08**
Nuts and nut products	*76·08*
Confectionery and cereal/nut/fruit/seed bars	**7·33**
Chocolate and chocolate-based confectionery	*3·97*
Muesli or cereal-style bars	*2·94*
Fruit, nut and seed bars	*0·42*
Cereals and cereal products	**5·21**
Breakfast cereals, ready to eat	*5·21*
Cereal-based products and dishes	**3·26**
Cakes, muffins, scones, cake-type desserts	*1·70*
Mixed dishes where cereal is the major ingredient	*0·79*
Sweet biscuits	*0·38*
Pastries	*0·38*
Savoury biscuits	*<0·01*
Fruit products and dishes	**3·13**
Dried fruit, preserved fruit	*2·58*
Mixed dishes where fruit is the major component	*0·55*
Vegetable products and dishes	**1·52**
Dishes where vegetable is the major component	*1·52*
Meat, poultry and game products and dishes	**1·26**
Mixed dishes where poultry or feathered game is the major component	*0·81*
Mixed dishes where beef, sheep, pork or mammalian game is the major component	*0·46*
Milk products and dishes	**0·87**
Frozen milk products	*0·51*
Flavoured milks and milkshakes	*0·36*
Cheese	*<0·01*
Savoury sauces and condiments	**0·63**
Dips	*0·47*
Gravies and savoury sauces	*0·16*
Sugar products and dishes	**0·53**
Jam and lemon spreads, chocolate spreads sauces	*0·53*
Non-alcoholic beverages	**0·07**
Fruit and vegetable juices and drinks	*0·07*
Fish and seafood products and dishes	**0·06**
Mixed dishes with fish or seafood as the major component	*0·06*
Snack foods	**0·05**
Other snacks	*0·05*

Major food groups shown in bold, sub-major food groups shown in italics.

* Day 1 data only (therefore, the number of observations differs from the number of nut consumers listed in Table 4, which was based on Days 1 and 2 data).

Within the major food group ‘Confectionery and cereal/nut/fruit/seed bars’, three sub-major food groups contributed to the nut intake of ‘nut consumers’. These were ‘Chocolate and chocolate-based confectionery’ (with an absolute contribution of 3·97 %), Muesli or cereal-style bars’ (2·94 %) and ‘Fruit, nut and seed bars’ (0·42 %) (Table 5). The ‘Cereals and cereal products’ major food group had only one sub-major food group contributing to the nut intake, called ‘Breakfast cereals, ready to eat’ (5·21 % of the total nut intake). Five sub-major food groups from the ‘Cereal-based products and dishes’ major food group contributed towards the nut intake of ‘nut consumers’. These were ‘Cakes, muffins, scones, cake-type desserts’ (1·70 %) and ‘Mixed dishes where cereal is the major ingredient’, ‘Sweet biscuits’, ‘Pastries’ and ‘Savoury biscuits’ (all contributing less than 1 %). In the Day 1 data of the 2011–2012 NNPAS, ‘nut consumers’ were consuming 88·25 % nuts (as a proportion of total nuts consumed) from non-discretionary products and 11·75 % of nut intake from discretionary products. The types of nuts that were most commonly reported by ‘nut consumers’ were peanuts (27·38 % of all nuts) and almonds (18·55 % of all nuts). ‘Nut consumers’ were obtaining 28·80 % of their nut intake from mixed nuts (i.e. those which included a combination of nut types), comprising 16·64 % of nut intake coming from mixed nuts containing peanuts and 12·16 % coming from mixed nuts without peanuts. Table 6 shows the proportions of each nut eaten by ‘nut consumers’ on Day 1 of the 2011–2012 NNPAS.

**Table 6:** Contribution of nut types towards the average nut intake of ‘nut consumers’, 2011–2012 National Nutrition and Physical Activity Survey (NNPAS) (number of observations: 3761)*

Nut type	Percentage of total nut intake
Mixed nuts	28·8
With peanut	16·64
Without peanut	12·16
Peanut	27·38
Almond	18·55
Cashew	12·12
Walnut	3·90
Macadamia	3·12
Pistachio	2·04
Hazelnut	1·29
Brazil nut	1·20
Chestnut	0·57
Pecan	0·50
Pine nut	0·46
Unknown	0·07

* Day 1 data only (therefore, the number of observations differs from the number of nut consumers listed in Table 4, which was based on Days 1 and 2 data).

## Discussion

To our knowledge, the current study is the first to report nut consumption from all food sources (including those found in mixed dishes and products) in a representative Australian sample (2011–2012 NNPAS). The current analysis was based on the application of a novel nut database. This research provides insights into nut consumption in Australia, as well as associations between intake of nuts and key nutrients, and anthropometric outcomes.

This research addressed gaps in the evidence base relating to nut consumption in Australia. The mean nut intake among Australians was 4·61 g/d. In those who reported consuming nuts (referred to as ‘nut consumers’, this was 11·75 g/d. The results of the current study show lower consumption figures to those previously available from the 2011–2012 NNPAS, which estimated the mean nut intake in Australia to be 5·2 g/d^(10)^. However, this latter figure emerged from an analysis based on a single major sub-group within the food database (‘Nuts and nut products’), and this includes peanuts, tree nuts, nut products and coconut and coconut products which would over-estimate total nut intake. In addition, it would not include nuts present in mixed dishes^(10)^. Furthermore, the 2011–2013 AHS analysis was based on data that captured a single day’s intake and this does not reflect usual intake, particularly for nuts which we know can be consumed sporadically. Nut consumption research conducted in New Zealand accounted for nuts found in mixed products and dishes, but only observed one day of dietary data, which does not reflect usual intake^(11)^. However, these studies, including our own, suggest that nut intake is considerably lower than dietary recommendations of 30 g of nuts per day^(26)^. This is concerning as the literature indicates that the risk of cardiovascular diseases, cancer and type 2 diabetes is lowered when nut consumption reaches 30 g/d^(1,26–29)^. In fact, the current study suggests that most of the Australian population and ‘nut consumers’ are consuming less than 10 g/d (Table 1). These results suggest that substantial increases in nut consumption are required to reach intakes associated with improved health outcomes.

Examining nut consumption according to demographic characteristics may be valuable in identifying groups at risk of low intakes. Differing patterns of nut consumption were observed across age groups and education levels. Adults aged 18–64 years had the highest intake of nuts, with children reporting the lowest intake of nuts. While this may reflect lower food intakes overall, additional analyses demonstrated the lowest proportion of ‘nut consumers’ in the child age group (data not shown). The lower proportion of ‘nut consumers’ in children could be due a range of factors including concerns regarding nut allergy and choking risk and schools or childcare centers prohibiting nuts and nut-containing products to reduce the risk of accidental exposure for young children with a nut allergy^(30)^. The literature has acknowledged the presence of nut allergy as reasons for low nut consumption^(31)^. It should be noted that current clinical guidance now recognises the importance of the early introduction of nuts for children at high risk of nut allergy^(32)^. The current Australian Dietary Guidelines recommend avoiding giving whole nuts to children under 3 years to minimise risk of choking and suggest textures need to be suitable (e.g. pastes) for younger children^(33)^. This restriction may in part contribute to the low nut intake levels in children. These factors, combined with the lower rates of nut consumption in this age group, suggest that nut consumption in children requires further investigation. Variation in nut consumption was also apparent between education levels. Among all Australians, the highest nut consumption was observed in those with higher level of education. These results align with those found in New Zealand, which found education level to be a significant predictor of consumption of total nuts^(11)^.

Nut consumption has previously been associated with an overall favourable dietary pattern^(7)^, and there is now an emerging body of evidence from intervention studies suggesting that the provision of nuts may facilitate the development of a higher quality diet^(34,35)^. The results of the current study align with these findings, with the consumption of nuts found to be associated with significantly higher intakes of a number of key nutrients, namely, fibre, vitamin E, Fe, Mg and P. A lack of significant association was found between nut consumption and Ca. There are a number of reasons why nut consumption may be associated with higher intakes of key nutrients. Nut intake may be consumed as a component of a healthier diet overall, which is also rich in foods such as fruit, vegetables and legumes such as the Mediterranean diet^(36)^. Consumption of nuts may facilitate consumption of other core foods, or lower consumption of discretionary foods, as previously shown by our team^(35)^.

Nuts are energy-dense foods, and both consumers and health professionals report concern regarding their impact on body weight^(31,37,38)^. As a result, it is pertinent to explore the relationship between nut consumption and anthropometric outcomes. Our results found that increased nut consumption was not associated with greater body weight, BMI or waist circumference, aligning with the broader body of evidence on this topic^(39)^. These findings suggest that despite their energy dense nature, higher consumption of nuts is not adversely associated with adiposity, which aligns with the findings of several intervention studies^(40)^. There are a number of mechanisms by which this may occur. Nuts are high in protein and satiating, and as a result may decrease the consumption of energy at subsequent meals^(41)^. The fat present in nuts is located in cell walls, meaning it is not readily absorbed, and with a proportion of the dietary fat instead excreted in the faeces^(42,43)^. As a result, the metabolisable energy available from nuts has been proposed to be 5–30 % lower than that estimated by Atwater factors^(44,45)^. Literature also suggests that people who consume nuts generally have a healthier diet and lifestyle^(5)^, which may in part explain these results. We considered this in our analysis by controlling for energy intake, age, sex and physical activity level to observe the association between nut intake and anthropometric outcomes. However, it is possible that other confounding variables (such as family history of overweight/obesity) were present which may have also affected results.

In addition to the results found for anthropometric variables, the relationship between nut consumption and blood pressure was examined. A lack of significant association was also found between nut consumption and blood pressure. This finding aligns with the findings of a recent meta-analysis^(20)^, although another analysis reported significant effects of nut consumption on systolic blood pressure in individuals without type 2 diabetes mellitus^(46)^. It should be noted that in contrast to these studies, the current analysis was a cross-sectional study, and therefore is only able to explore the relationship between nut consumption and health outcomes. Further exploration of the effect of nut intake on blood pressure through randomised controlled trials is required.

In addition to identifying the total amount of nuts consumed, examining all the food sources of nuts can be useful in understanding consumption patterns and informing dietetic strategies to increase nut intake. In particular, it is important to identify how much of the total nut intake is from discretionary sources, given the 2013 Australian Dietary Guidelines recommend limiting consumption of discretionary foods and given approximately 35 % of energy within Australian diets comes from discretionary foods^(10)^. While whole nuts are considered to be a core food, they may be consumed as part of a mixed discretionary food. In the current analysis, almost 90 % of nut consumption was found to come from core food groups, including when nuts were consumed as whole nuts. The core nut-containing foods within the 2011–2012 NNPAS included almonds, cashews and walnuts, among others, while nut-containing discretionary products included ice-creams, chocolate-coated nuts and muesli bars. Discretionary food products, while advised to be consumed only occasionally, are convenient and can add variety to an individual’s diet. Although it is encouraging to note that the majority of nuts consumed were in the form of core foods, given the relationship between discretionary foods and chronic disease, future research could explore the discretionary sources of nuts. In turn, dietary advice may address the sources of nut consumption to encourage consumption of the core sources of nuts while limiting the consumption of discretionary sources. In addition, most of the nuts consumed were from the ‘Seed and nut products and dishes: ‘Nuts and nut products’ sub-major food group, indicating more consumption of nuts as a core food rather than within discretionary products. The second highest level of nuts was consumed from the ‘Confectionery and cereal/nut/fruit/seed bars’ group which includes nut-containing products such as muesli bars, peanut brittle and chocolate bars. The presence of nuts in multiple food groups highlights the importance of the current study, as nuts are found in a wide range of products that previously were not accounted for in nutrition research.

The current study was not without limitations. The 2011–2012 NNPAS collected dietary data using the 24-h recall method, which are susceptible to recall bias as participants may forget to recall food and beverages consumed or may over-report or under-report foods to conform to societal expectations. In particular, while self-reported Na intake was included in the analyses exploring associations of nut intake with blood pressure, dietary assessment of Na is known to be flawed^(47)^. When reporting nut consumption, all participants in the 2011–2012 NNPAS were included regardless of whether they were avoiding consuming nuts, for example, due to allergies. Although this approach was appropriate given the purpose of the current analysis was to report nut consumption in Australia, it should be noted when interpreting results. To ensure that nut intakes could be compared with population recommendations (which are given in g/d), consumption of nuts was reported in grams rather than as a percentage of energy. While this aligns with the format of population recommendations, it should be noted that comparison of intakes across age and gender groups would likely have been affected by overall energy intakes. To provide an overview of contributions to food group and nutrient intakes, analyses of sources of nut intake and contributions to nutrient intakes were required to be limited to Day 1 data only. Due to the structuring of education level categories, which were used in the regression models, individuals currently attending school (i.e. children) were excluded from these models, meaning the associations between nut consumption, nutrient intake, anthropometric outcomes and blood pressure were performed for adults only. Finally, people who consume nuts may be more likely to value their health and lead a healthier lifestyle. This could include having a healthier diet overall and participating in regular physical activity among other factors, meaning nut intake may be merely a marker of a healthier lifestyle which leads to a lower weight or BMI, rather than the driving factor. We have modelled the proposed relationship between nut consumption and anthropometric, blood pressure and nutrient outcomes; however, as with all analyses, it is possible that there are other unknown confounding variables which were not present in the model.

## Conclusion

The analysis presented here is the first to explore nut consumption in a representative Australian sample which accounts for nuts from all sources. The results indicate that both the Australian population overall, and ‘nut consumers’ as a sub-group in that population, are not consuming the recommendation of 30 g of nuts per day. This is despite the literature demonstrating health benefits from consuming this amount. The results of the current analysis support the current literature base, whereby increasing nut consumption is not associated with higher body weight, BMI or waist circumference, despite their energy dense nature. Nut consumption was also associated with higher intakes of a number of key nutrients. In future, strategies to increase nut intake could focus on the relationship between nut consumption and health outcomes and the need to include recommended levels of about 30 g of nuts per day in a healthy diet.

## Supplementary material

For supplementary material accompanying this paper visit https://doi.org/10.1017/S1368980019004117. click here to view supplementary material
