# Supplementary material for: Nut consumption in a representative survey of Australians: a secondary analysis of the 2011–2012 National Nutrition and Physical Activity Survey
Source: Public Health Nutr. 2020 Mar 10;23(18):3368–78. doi: 10.1017/S1368980019004117 (PMC7737041; doi:10.1017/S1368980019004117)
Supplement: Supplementary file 1 [file S1368980019004117sup001.docx]

#### Supplementary material 1: Directed acyclic graph demonstrating proposed causal model underlying the linear regression between nut consumption and nutrient intakes.

####
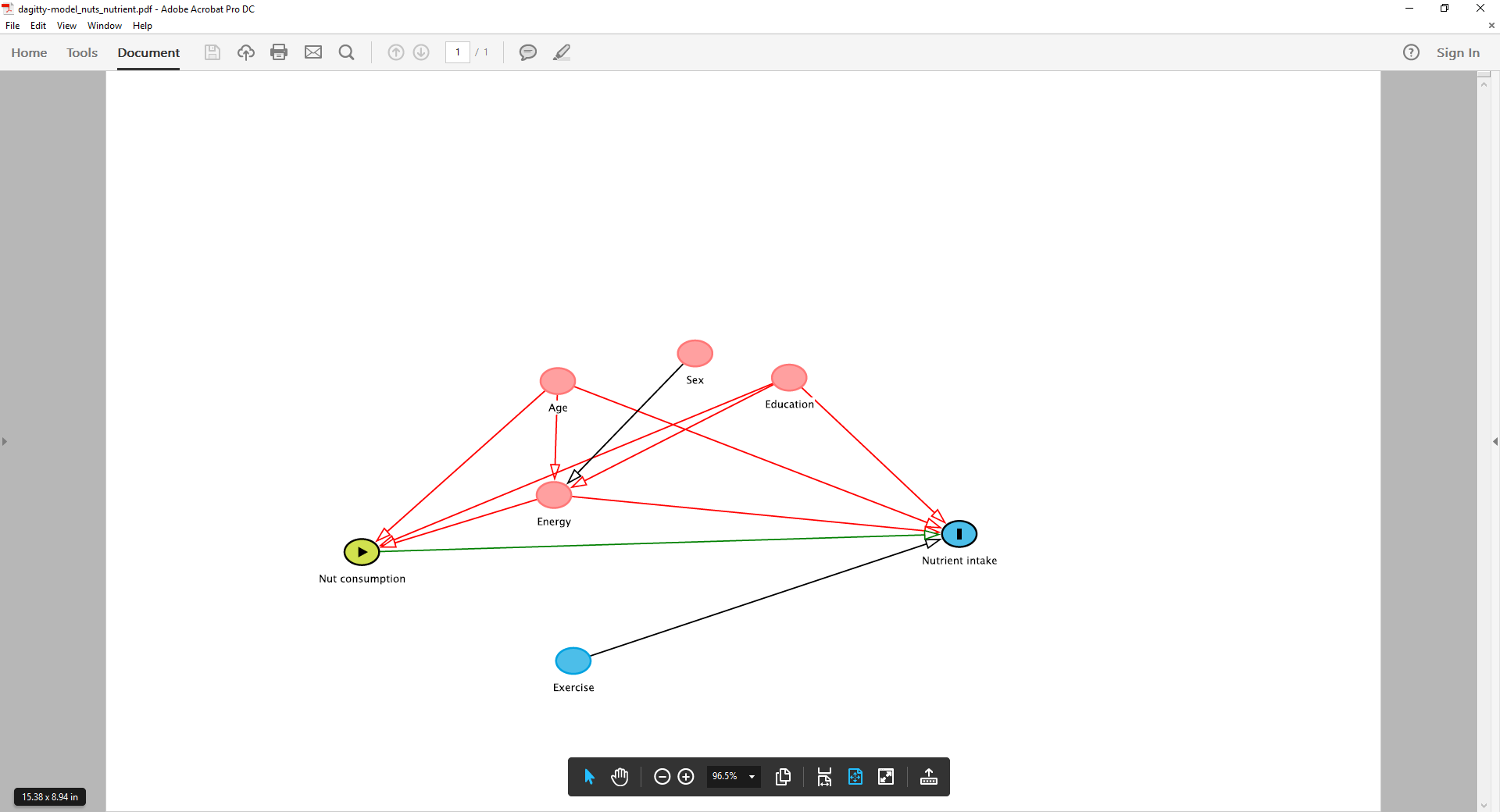


#### Supplementary material 2: Linear regression between nut consumption (g) and fibre (g) intake in the 2011-12 NNPAS. (population size: 16,598,770, number of observations: 9,188)

|  | **Coefficient** | **T** | **P>\|t\|** | **95% confidence interval** |
| --- | --- | --- | --- | --- |
| Nuts (g) | 0.090 | 8.890 | <0.001 | 0.070, 0.110 |
| Energy (kJ) | 0.002 | 40.820 | <0.001 | 0.002, 0.002 |
| *Exercise level*^1^ | |  |  |  |
| 1 | 1.951 | 5.250 | <0.001 | 1.208, 2.695 |
| 2 | 1.211 | 5.070 | <0.001 | 0.732, 1.689 |
| 3 | 0.756 | 3.620 | 0.001 | 0.337, 1.174 |
| 4 | 0.408 | 0.530 | 0.597 | -1.128, 1.945 |
| *Sex* | |  |  |  |
| Female | 1.296 | 6.470 | <0.001 | 0.896, 1.697 |
| Age (years) | 0.074 | 14.930 | <0.001 | 0.064, 0.084 |
| *Highest level of non-school education*^2^ | |  |  |  |
| 1 | 0.914 | 2.690 | 0.009 | 0.233, 1.595 |
| 2 | 1.070 | 3.910 | <0.001 | 0.522, 1.617 |
| 3 | 0.543 | 1.800 | 0.077 | -0.061, 1.147 |
| 4 | -0.183 | -0.800 | 0.428 | -0.640, 0.275 |
| 5 | -0.473 | -1.240 | 0.222 | -1.239, 0.293 |
| 6 | -1.243 | -1.850 | 0.069 | -2.585, 0.099 |

^1^Measured in ‘levels’ where Level 1 indicates ‘high’ level of physical activity and Level 5 indicates ‘sedentary’ activity.

^2^Measured in ‘levels’ where Level 1 indicates highest level of non-school education, and Level 7 indicates lowest level

####

**Supplementary material 3:** Linear regression between nut consumption (g) and vitamin E intake (mg) in the 2011-12 NNPAS. (population size: 16,598,770, number of observations: 9,188)

|  | **Coefficient** | **T** | **P>\|t\|** | **95% confidence interval** |
| --- | --- | --- | --- | --- |
| Nuts (g) | 0.070 | 11.420 | <0.001 | 0.058, 0.082 |
| Energy (kJ) | 0.001 | 39.150 | <0.001 | 0.001, 0.001 |
| *Exercise level*^1^ | |  |  |  |
| 1 | 0.912 | 6.300 | <0.001 | 0.622, 1.202 |
| 2 | 0.418 | 4.280 | <0.001 | 0.222, 0.614 |
| 3 | 0.223 | 2.240 | 0.029 | 0.024, 0.422 |
| 4 | 0.070 | 0.230 | 0.820 | -0.540, 0.680 |
| *Sex* | |  |  |  |
| female | 0.853 | 9.800 | <0.001 | 0.679, 1.027 |
| Age (years) | 0.009 | 4.520 | <0.001 | 0.005, 0.013 |
| *Highest level of non-school education*^2^ | |  |  |  |
| 1 | 0.495 | 4.150 | <0.001 | 0.257, 0.734 |
| 2 | 0.627 | 5.390 | <0.001 | 0.394, 0.860 |
| 3 | 0.511 | 4.010 | <0.001 | 0.256, 0.860 |
| 4 | 0.149 | 1.400 | 0.167 | -0.064, 0.361 |
| 5 | 0.169 | 1.190 | 0.241 | -0.116, 0.454 |
| 6 | 0.589 | 1.490 | 0.142 | -0.204, 1.382 |

^1^Measured in ‘levels’ where Level 1 indicates ‘high’ level of physical activity and Level 5 indicates ‘sedentary’ activity.

^2^Measured in ‘levels’ where Level 1 indicates highest level of non-school education, and Level 7 indicates lowest level

Supplementary material 4: Linear regression between nut consumption (g) and iron intake (mg) in the 2011-12 NNPAS (population size: 16,598,770, number of observations: 9,188)

|  | **Coefficient** | **T** | **P>\|t\|** | **95% confidence interval** |
| --- | --- | --- | --- | --- |
| Nuts (g) | 0.014 | 3.570 | 0.001 | 0.006, 0.023 |
| Energy (kJ) | 0.001 | 40.360 | <0.001 | 0.001, 0.001 |
| *Exercise level*^1^ |  |  |  |  |
| 1 | 0.865 | 6.250 | <0.001 | 0.588, 1.142 |
| 2 | 0.363 | 3.000 | 0.004 | 0.121, 0.605 |
| 3 | 0.328 | 3.090 | 0.003 | 0.115, 0.541 |
| 4 | 0.311 | 0.820 | 0.418 | -0.452, 1.074 |
| *Sex* |  |  |  |  |
| female | -0.034 | -0.420 | 0.677 | -0.194, 0.127 |
| Age (years) | 0.020 | 9.100 | <0.001 | 0.015, 0.024 |
| *Highest level of non-school education*^2^ |  |  |  |  |
| 1 | 0.067 | 0.490 | 0.624 | -0.204, 0.337 |
| 2 | 0.088 | 0.800 | 0.426 | -0.131, 0.307 |
| 3 | 0.029 | 0.260 | 0.794 | -0.195, 0.253 |
| 4 | -0.007 | -0.070 | 0.945 | -0.211, 0.197 |
| 5 | -0.155 | -0.850 | 0.400 | -0.520, 0.210 |
| 6 | -0.743 | -2.760 | 0.008 | -1.282, -0.204 |

^1^Measured in ‘levels’ where Level 1 indicates ‘high’ level of physical activity and Level 5 indicates ‘sedentary’ activity.

^2^Measured in ‘levels’ where Level 1 indicates highest level of non-school education, and Level 7 indicates lowest level

Supplementary material 5: Linear regression between nut consumption (g) and magnesium intake (mg) in the 2011-12 NNPAS (population size: 16,598,770, number of observations: 9,188)

|  | **Coefficient** | **T** | **P>\|t\|** | **95% confidence interval** |
| --- | --- | --- | --- | --- |
| Nuts (g) | 1.825 | 14.430 | <0.001 | 1.572, 2.078 |
| Energy (kJ) | 0.030 | 61.410 | <0.001 | 0.030, 0.031 |
| *Exercise level*^1^ |  |  |  |  |
| 1 | 40.161 | 11.750 | <0.001 | 33.324, 46.998 |
| 2 | 18.029 | 7.630 | <0.001 | 13.297, 22.760 |
| 3 | 8.988 | 4.000 | <0.001 | 4.487, 13.489 |
| 4 | 1.433 | 0.190 | 0.852 | -13.910, 16.776 |
| *Sex* |  |  |  |  |
| female | 9.284 | 4.910 | <0.001 | 5.504, 13.064 |
| Age (years) | 0.628 | 11.470 | <0.001 | 0.519, 0.738 |
| *Highest level of non-school education*^2^ |  |  |  |  |
| 1 | 17.882 | 5.060 | <0.001 | 10.808, 24.956 |
| 2 | 17.066 | 5.730 | <0.001 | 11.105, 23.028 |
| 3 | 14.908 | 5.000 | <0.001 | 8.941, 20.875 |
| 4 | 7.276 | 2.670 | 0.010 | 1.818, 12.733 |
| 5 | 7.172 | 1.880 | 0.065 | -0.455, 14.798 |
| 6 | -12.183 | -2.160 | 0.035 | -23.450, -0.917 |

^1^Measured in ‘levels’ where Level 1 indicates ‘high’ level of physical activity and Level 5 indicates ‘sedentary’ activity.

^2^Measured in ‘levels’ where Level 1 indicates highest level of non-school education, and Level 7 indicates lowest level

Supplementary material 6: Linear regression between nut consumption (g) and phosphorous intake (mg) in the 2011-12 NNPAS (population size: 16,598,770, number of observations: 9,188)

|  | **Coefficient** | **T** | **P>\|t\|** | **95% confidence interval** |
| --- | --- | --- | --- | --- |
| Nuts (g) | 0.709 | 2.200 | 0.032 | 0.064, 1.354 |
| Energy (kJ) | 0.140 | 61.500 | <0.001 | 0.136, 0.145 |
| *Exercise level*^1^ |  |  |  |  |
| 1 | 95.887 | 9.600 | <0.001 | 75.892, 115.883 |
| 2 | 34.025 | 4.110 | <0.001 | 17.451, 50.599 |
| 3 | 17.874 | 2.200 | 0.032 | 1.608, 34.141 |
| 4 | 26.556 | 0.520 | 0.607 | -76.188, 129.300 |
| *Sex* |  |  |  |  |
| female | 17.865 | 2.640 | 0.010 | 4.343, 31.388 |
| Age (years) | 1.517 | 7.910 | <0.001 | 1.133, 1.901 |
| *Highest level of non-school education*^2^ |  |  |  |  |
| 1 | 13.373 | 1.070 | 0.288 | -11.607, 38.354 |
| 2 | 9.553 | 1.030 | 0.308 | -9.042, 28.147 |
| 3 | 10.971 | 1.200 | 0.235 | -7.328, 29.270 |
| 4 | 14.625 | 1.550 | 0.127 | -4.298, 33.549 |
| 5 | 18.248 | 1.320 | 0.192 | -9.428, 45.924 |
| 6 | -36.981 | -0.940 | 0.349 | -115.297, 41.335 |

^1^Measured in ‘levels’ where Level 1 indicates ‘high’ level of physical activity and Level 5 indicates ‘sedentary’ activity.

^2^Measured in ‘levels’ where Level 1 indicates highest level of non-school education, and Level 7 indicates lowest level

Supplementary material 7: Linear regression between nut consumption (g) and calcium intake (mg) in the 2011-12 NNPAS (population size: 16,598,770, number of observations: 9,188)

|  | **Coefficient** | **T** | **P>\|t\|** | **95% confidence interval** |
| --- | --- | --- | --- | --- |
| Nuts (g) | -0.129 | -0.400 | 0.689 | -0.768, 0.511 |
| Energy (kJ) | 0.073 | 37.030 | <0.001 | 0.069, 0.077 |
| *Exercise level*^1^ |  |  |  |  |
| 1 | 69.101 | 5.820 | <0.001 | 45.323, 92.878 |
| 2 | 14.998 | 1.750 | 0.085 | -2.155, 32.152 |
| 3 | 9.305 | 1.230 | 0.225 | -5.872, 24.483 |
| 4 | 16.435 | 0.470 | 0.642 | -54.010, 86.881 |
| *Sex* |  |  |  |  |
| female | 55.781 | 9.500 | <0.001 | 44.034, 67.528 |
| Age (years) | 0.315 | 1.820 | 0.073 | -0.030, 0.661 |
| *Highest level of non-school education*^2^ |  |  |  |  |
| 1 | 19.165 | 1.510 | 0.137 | -6.264, 44.595 |
| 2 | 25.471 | 2.980 | 0.004 | 8.372, 42.570 |
| 3 | 21.431 | 2.090 | 0.041 | 0.869, 41.993 |
| 4 | 9.930 | 0.890 | 0.375 | -12.278, 32.137 |
| 5 | 25.522 | 2.060 | 0.044 | 0.749, 50.295 |
| 6 | -34.541 | -1.040 | 0.304 | -101.168, 32.085 |

^1^Measured in ‘levels’ where Level 1 indicates ‘high’ level of physical activity and Level 5 indicates ‘sedentary’ activity.

^2^Measured in ‘levels’ where Level 1 indicates highest level of non-school education, and Level 7 indicates lowest level

#### Supplementary material 8: Directed acyclic graph demonstrating proposed causal model underlying the linear regression between nut consumption and anthropometric outcomes.

####
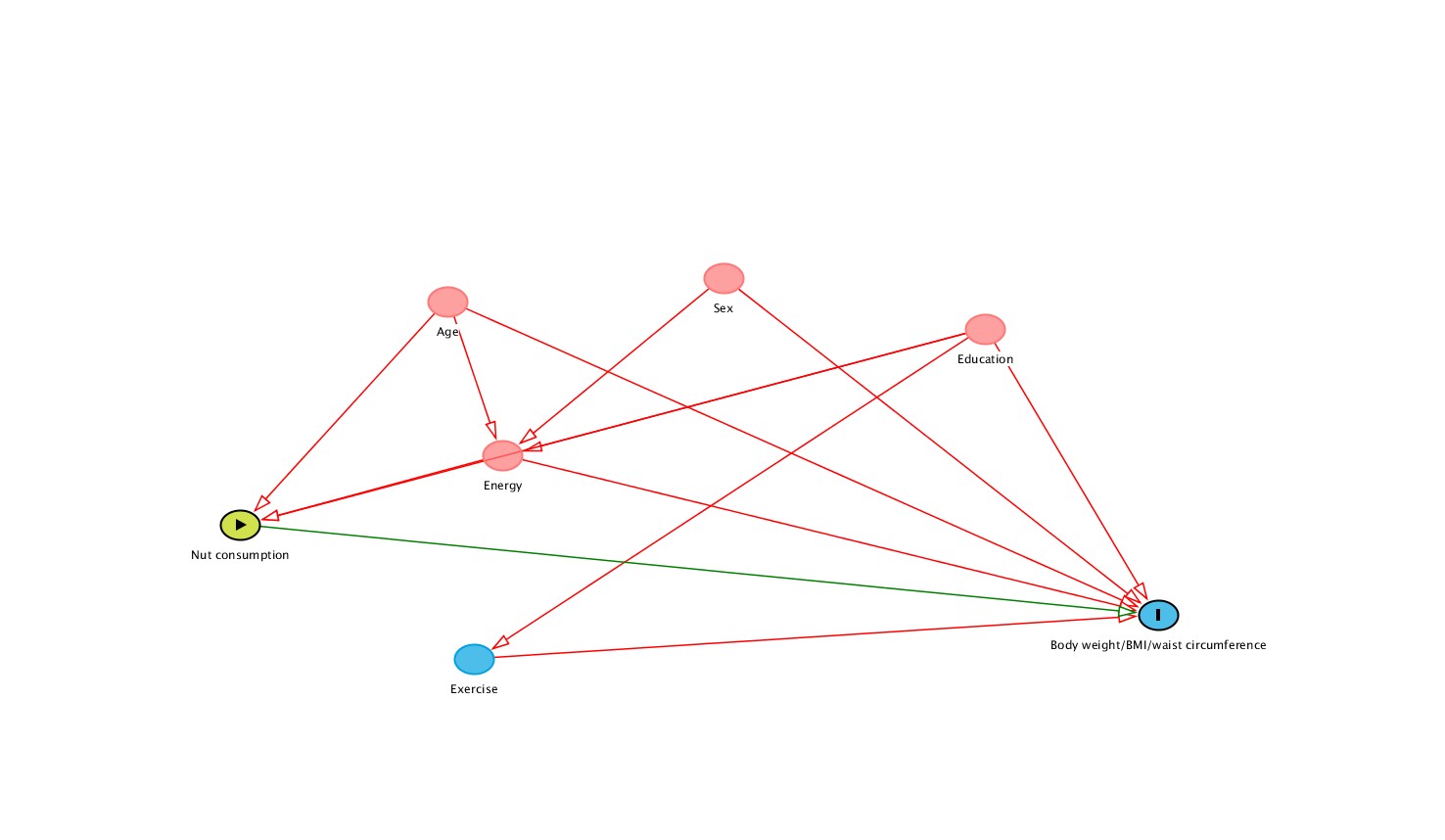


#### Supplementary material 9: Directed acyclic graph demonstrating proposed causal model underlying the linear regression between nut consumption and blood pressure

####
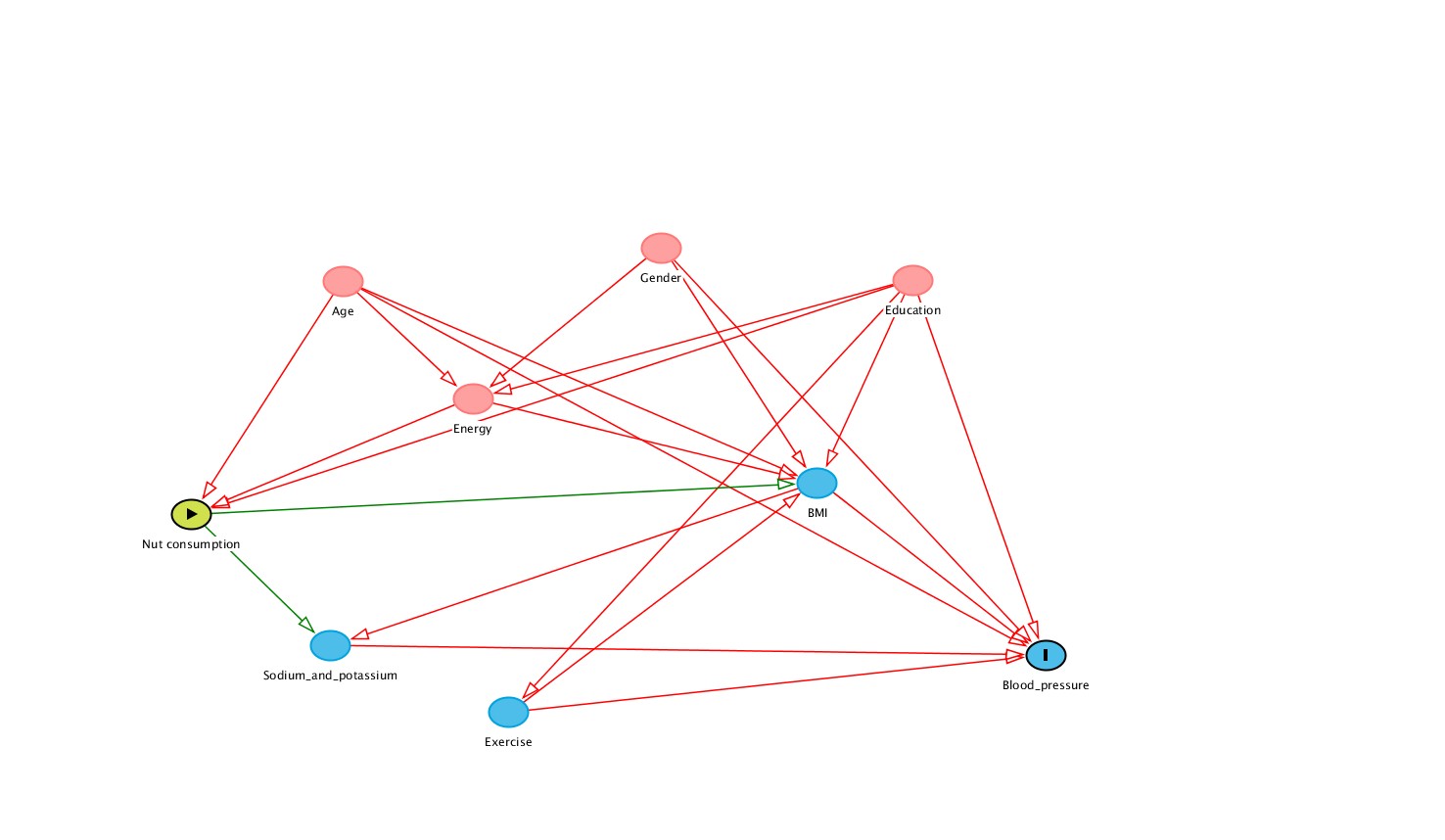


Supplementary material 10: Linear regression between nut consumption (g) and body weight (kg) in the 2011-12 NNPAS (population size: 14,288,226, number of observations: 7,800)

|  | **Coefficient** | **T** | **P>\|t\|** | **95% confidence interval** |
| --- | --- | --- | --- | --- |
| Nuts (g) | -0.006 | -0.220 | 0.823 | -0.061, 0.049 |
| Energy (kJ) | -0.000 | -1.120 | 0.269 | -0.001, 0.000 |
| *Exercise level*^1^ |  |  |  |  |
| 1 | -3.266 | -3.170 | 0.002 | -5.330, -1.202 |
| 2 | -2.235 | -2.540 | 0.014 | -3.997, -0.474 |
| 3 | -2.052 | -2.520 | 0.015 | -3.684, -0.421 |
| 4 | -1.844 | -0.730 | 0.470 | -6.916, 3.228 |
| *Sex* |  |  |  |  |
| female | -14.437 | -27.150 | <0.001 | -15.501, -13.373 |
| Age (years) | 0.050 | 3.630 | 0.001 | 0.023, 0.078 |
| *Highest level of non-school education*^2^ |  |  |  |  |
| 1 | -0.118 | -0.120 | 0.908 | -2.156, 1.920 |
| 2 | -1.729 | -2.330 | 0.023 | -3.215, -0.242 |
| 3 | -0.247 | -0.310 | 0.761 | -1.864, 1.370 |
| 4 | 2.604 | 3.510 | 0.001 | 1.118, 4.089 |
| 5 | -0.851 | -0.840 | 0.407 | -2.889, 1.187 |
| 6 | 5.249 | 2.370 | 0.021 | 0.814, 9.685 |

^1^Measured in ‘levels’ where Level 1 indicates ‘high’ level of physical activity and Level 5 indicates ‘sedentary’ activity.

^2^Measured in ‘levels’ where Level 1 indicates highest level of non-school education, and Level 7 indicates lowest level

Supplementary material 11: Linear regression between nut consumption (g) and BMI (kg/m^2^) in the 2011-12 NNPAS (population size: 14,200,356, number of observations: 7,751)

|  | **Coefficient** | **T** | **P>\|t\|** | **95% confidence interval** |
| --- | --- | --- | --- | --- |
| Nuts (g) | -0.007 | -0.870 | 0.390 | -0.024, 0.010 |
| Energy (kJ) | -0.000 | -3.140 | 0.003 | -0.000, -0.000 |
| *Exercise level*^1^ |  |  |  |  |
| 1 | -1.549 | -4.950 | <0.001 | -2.176, -0.923 |
| 2 | -0.989 | -3.800 | <0.001 | -1.510, -0.468 |
| 3 | -0.745 | -2.770 | 0.007 | -1.284, -0.207 |
| 4 | -1.105 | -1.200 | 0.233 | -2.942, 0.732 |
| *Sex* |  |  |  |  |
| female | -0.823 | -5.010 | <0.001 | -1.151, -0.494 |
| Age (years) | 0.058 | 12.460 | <0.001 | 0.048, 0.067 |
| *Highest level of non-school education*^2^ |  |  |  |  |
| 1 | -0.297 | -0.900 | 0.374 | -0.962, 0.367 |
| 2 | -0.836 | -3.670 | 0.001 | -1.292, -0.380 |
| 3 | -0.378 | -1.480 | 0.145 | -0.891, 0.134 |
| 4 | 0.663 | 2.740 | 0.008 | 0.178, 1.148 |
| 5 | -0.353 | -1.030 | 0.307 | -1.038, 0.332 |
| 6 | 1.507 | 2.200 | 0.031 | 0.139, 2.876 |

^1^Measured in ‘levels’ where Level 1 indicates ‘high’ level of physical activity and Level 5 indicates ‘sedentary’ activity.

^2^Measured in ‘levels’ where Level 1 indicates highest level of non-school education, and Level 7 indicates lowest level

Supplementary material 12: Linear regression between nut consumption (g) and waist circumference (cm) in the 2011-12 NNPAS (population size: 14,075,179, number of observations: 7,733)

|  | **Coefficient** | **T** | **P>\|t\|** | **95% confidence interval** |
| --- | --- | --- | --- | --- |
| Nuts (g) | -0.028 | -1.350 | 0.181 | -0.069, 0.013 |
| Energy (kJ) | -0.000 | -1.290 | 0.203 | -0.000, 0.000 |
| *Exercise level*^1^ |  |  |  |  |
| 1 | -6.063 | -7.570 | <0.001 | -7.666, -4.461 |
| 2 | -3.038 | -4.740 | <0.001 | -4.322, -1.755 |
| 3 | -2.136 | -3.550 | 0.001 | -3.339, -0.934 |
| 4 | -1.903 | -0.820 | 0.417 | -6.562, 2.757 |
| *Sex* |  |  |  |  |
| female | -10.577 | -25.070 | <0.001 | -11.421, -9.732 |
| Age (years) | 0.228 | 19.620 | <0.001 | 0.205, 0.251 |
| *Highest level of non-school education*^2^ |  |  |  |  |
| 1 | -0.540 | -0.670 | 0.504 | -2.149, 1.068 |
| 2 | -1.974 | -3.450 | 0.001 | -3.119, -0.829 |
| 3 | -0.525 | -0.820 | 0.417 | -1.810, 0.760 |
| 4 | 1.406 | 2.480 | 0.016 | 0.272, 2.541 |
| 5 | -0.177 | -0.170 | 0.868 | -2.312, 1.957 |
| 6 | 3.498 | 1.760 | 0.083 | -0.469, 7.464 |

^1^Measured in ‘levels’ where Level 1 indicates ‘high’ level of physical activity and Level 5 indicates ‘sedentary’ activity.

^2^Measured in ‘levels’ where Level 1 indicates highest level of non-school education, and Level 7 indicates lowest level

Supplementary material 13: Linear regression between nut consumption (g) and systolic blood pressure (mmHg) in the 2011-12 NNPAS (population size: 13,712,136, number of observations: 7,505)

|  | **Coefficient** | **T** | **P>\|t\|** | **95% confidence interval** |
| --- | --- | --- | --- | --- |
| Nuts (g) | -0.006 | -0.240 | 0.810 | -0.055, 0.043 |
| Energy (kJ) | 0.000 | 1.880 | 0.065 | -0.000, 0.001 |
| Sodium (mg) | 0.000 | 0.360 | 0.719 | -0.001, 0.001 |
| Potassium (mg) | -0.001 | -1.680 | 0.098 | -0.002, 0.000 |
| *Exercise level*^1^ |  |  |  |  |
| 1 | 1.379 | 1.450 | 0.152 | -0.524, 3.282 |
| 2 | 0.189 | 0.240 | 0.812 | -1.392, 1.770 |
| 3 | 0.146 | 0.190 | 0.852 | -1.414, 1.706 |
| 4 | 6.164 | 2.310 | 0.024 | 0.829, 11.499 |
| *Sex* |  |  |  |  |
| female | -5.440 | -11.120 | <0.001 | -6.419, -4.461 |
| Age (years) | 0.471 | 29.450 | <0.001 | 0.439, 0.503 |
| BMI (kg/m^2^) | 0.542 | 8.990 | <0.001 | 0.421, 0.662 |
| *Highest level of non-school education*^2^ |  |  |  |  |
| 1 | -2.651 | -2.690 | 0.009 | -4.619, -0.682 |
| 2 | -1.926 | -2.220 | 0.030 | -3.658, -0.194 |
| 3 | -1.802 | -2.130 | 0.038 | -3.497, -0.108 |
| 4 | -1.613 | -2.260 | 0.027 | -3.039, -0.187 |
| 5 | 1.632 | 1.230 | 0.225 | -1.033, 4.296 |
| 6 | -2.592 | -1.280 | 0.206 | -6.646, 1.461 |

^1^Measured in ‘levels’ where Level 1 indicates ‘high’ level of physical activity and Level 5 indicates ‘sedentary’ activity.

^2^Measured in ‘levels’ where Level 1 indicates highest level of non-school education, and Level 7 indicates lowest level

Supplementary material 14: Linear regression between nut consumption and diastolic blood pressure in the 2011-12 NNPAS (population size: 13,712,136, number of observations: 7,505)

|  | **Coefficient** | **T** | **P>\|t\|** | **95% confidence interval** |
| --- | --- | --- | --- | --- |
| Nuts (g) | 0.007 | 0.430 | 0.671 | -0.027, 0.041 |
| Energy (kJ) | 0.000 | 0.140 | 0.886 | -0.000, 0.000 |
| Sodium (mg) | -0.000 | 0.000 | 0.996 | -0.001, 0.001 |
| Potassium (mg) | -0.000 | -0.640 | 0.522 | -0.001, 0.001 |
| *Exercise level*^1^ |  |  |  |  |
| 1 | -1.896 | -3.400 | 0.001 | -3.011, -0.780 |
| 2 | -0.218 | -0.440 | 0.661 | -1.206, 0.771 |
| 3 | -0.352 | -0.760 | 0.448 | -1.272, 0.569 |
| 4 | 0.798 | 0.440 | 0.658 | -2.792, 4.389 |
| *Sex* |  |  |  |  |
| female | -0.913 | -2.980 | 0.004 | -1.526, -0.300 |
| Age (years) | 0.065 | 7.490 | <0.001 | 0.048, 0.083 |
| BMI (kg/m^2^) | 0.629 | 17.600 | <0.001 | 0.558, 0.701 |
| *Highest level of non-school education*^2^ |  |  |  |  |
| 1 | 2.386 | 3.440 | 0.001 | 0.997, 3.776 |
| 2 | 1.425 | 2.510 | 0.015 | 0.287, 2.563 |
| 3 | 0.622 | 1.080 | 0.286 | -0.535, 1.778 |
| 4 | 0.569 | 1.090 | 0.280 | -0.475, 1.614 |
| 5 | 0.759 | 0.770 | 0.444 | -1.211, 2.728 |
| 6 | -0.124 | -0.130 | 0.896 | -2.015, 1.766 |

^1^Measured in ‘levels’ where Level 1 indicates ‘high’ level of physical activity and Level 5 indicates ‘sedentary’ activity.

^2^Measured in ‘levels’ where Level 1 indicates highest level of non-school education, and Level 7 indicates lowest level
